# Flexion Dysfunction of Atlanto‐Occipital Joint Associated with Cervical Spondylosis

**DOI:** 10.1111/os.12928

**Published:** 2021-01-15

**Authors:** Long Gong, Hao‐ning Ma, Ping Yi, Ming‐sheng Tan

**Affiliations:** ^1^ Department of Orthopaedic China‐Japan Friendship Hospital, Peking Union Medical College, Chinese Academy of Medical College Beijing China

**Keywords:** Atlanto‐occipital joint, Cervical spondylosis, Flexion dysfunction

## Abstract

**Objective:**

To investigate the association between atlanto‐occipital radiographic alignment in flexion and cervical spondylosis (CS).

**Methods:**

This is a retrospective case‐control study. CS patients were recruited from our hospital, and the age/gender/body mass index (BMI)‐matched healthy controls were selected from the subjects in health examinations at the same hospital between January 2015 and May 2019. A total of 464 subjects was included in the study. There are 282 males and 182 females. The ages of patients were 20 to 67 years, and the mean age was 33.9 years. CS patients were considered the case group. Based on surgical treatments, they were subdivided into non‐operation group and operation group. The operation group and non‐operation group had 45 and 187 patients, respectively, while 232 subjects were included in the control group. The angle between McGregor's line and C_1_ line (O‐C_1_ angle) was evaluated on images taken in flexion (F‐OC) and neutral positions (N‐OC) independently. The relationship between the FOC (FOC=F‐OC—N‐OC) and Neck Disability Index (NDI) was examined, and the involvement of the FOC in the onset of CS was analyzed. Receiver operating characteristic (ROC) curve analysis was performed to determine the optimal cut‐off for detecting an increased risk of CS.

**Results:**

The median follow‐up time was 51.6 months (25–115 months). The case groups, especially the operation group, tended to be older (55.8 ± 11.2 *vs* 41.6 ± 13.8 *vs* 23.5 ± 5.5 years, *P* < 0.001), have a higher NDI score (12.2 ± 4.5 *vs* 6.2 ± 2.1 *vs* 3.2 ± 1.2, *P* < 0.001), and longer medical history (10.5 ± 9.5 *vs* 6.8 ± 11.2 years, *P* < 0.001). One‐way analysis of variance showed statistically significant differences in FOC between the control and case groups (1.4° ± 1.2° *vs* 3.6° ± 1.9° *vs* 7.2° ± 2.0°, *P* < 0.001). Besides, a post‐hoc Tukey test showed a lower FOC in the operation group compared with that in the non‐operation group (1.4° ± 1.2° *vs* 3.6° ± 1.9°, *P* < 0.001). Using FOC as a radiological predictive model to predict CS, the cut‐off value was 4.2°. Using FOC as a radiological predictive model to predict CS, the area under the curve (AUC) was 0.86 (95% *CI*: 0.78–0.92, *P* < 0.001). In the univariable risk analysis model, conditional logistic regression showed that the FOC level was an independent factor with an important role in the risk of CS. The odds rose to 8.2 times when FOC reached the level under 4.2° (*OR* = 8.2; 95% *CI*: 6.4–10.0; *P* < 0.001). There existed a significant negative correlation between FOC levels and NDI (*r* = −0.451, *P* = 0.016).

**Conclusions:**

Stiff O‐C_1_, which is defined as FOC ≤ 4.2°, represented decreased flexion dysfunction of atlanto‐occipital joint and is closely associated with high risk for the occurrence of CS. This finding could show a possible relationship between upper and lower cervical spine and help spine surgeons to understand the pathological process of CS and implement appropriate management.

## Graphical abstract

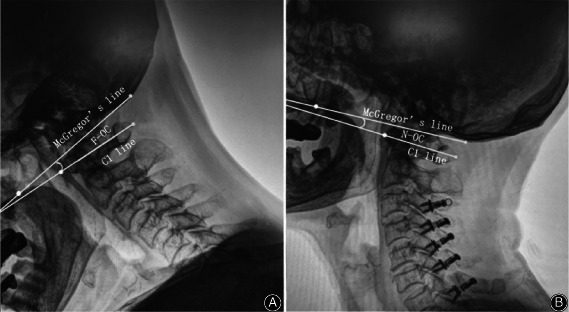

FOC less than 4.2° could experience an 8.2‐fold increase in the odds of developing cervical spondylosis (CS).

## Introduction

Cervical spondylosis (CS) is not considered a rare condition, with a reported prevalence of 5% to 21%^1^, ^2^, ^3^. Degenerative changes of the cervical spine are seen radiographically in over half of the population aged 55 years or higher^2^. Rates of surgery for this condition increased by 90% in the US population from 1990 to 2000^2^. With an increasingly sedentary population, especially with reliance on mobile devices, the current prevalence rate can be higher^3^. The pathogenesis of CS has been convincingly related to primary disc degeneration, disc space reduction, and subsequent pathological processes, such as osteophyte formation and ligament flavum hypertrophy, that eventually lead to spinal and neural canal stenosis and related neurological symptoms and deficits^1^, ^2^. Symptoms caused by CS can be categorized broadly into three clinical syndromes: axial neck pain, cervical radiculopathy, and cervical myelopathy; with patients commonly having a combination of these syndromes. A previous study has suggested that the changes of structure and function of cervical disc with degeneration is related to neck pain^2^, ^4^. This neck pain is usually accompanied by stiffness of the neck, headache, unilateral or bilateral shoulder pain, non‐root arm pain, ocular and vestibular dysfunction, and pain in the anterior chest wall^3^. Unfortunately, currently CS is quite common among young people, and even teenagers who are generally expected to be far from degenerative processes due to their age^3^. In this context, the question arises: what pushes them to suffer these pathological changes early?

Previous studies revealed that the abnormal atlantoaxial joint accelerates spinal degeneration as it involves the disorder of the muscular–ligament balance system and mechanical stress conduction^4^, ^5^, ^6^, ^7^. The atlantoaxial joint allows for flexion and extension of 15° and 30° of axial rotation^7^. In contrast, the atlanto‐occipital joint accommodates 25° of flexion and extension and 5° of axial rotation^7^. Significant relationships between the alignments of the upper and subaxial cervical spine have been clearly established in asymptomatic subjects^4^, ^8^. Several studies demonstrated that atlantoaxial instability can result in compensatory change in subaxial alignment of cervical spine and further lead to spinal degeneration like osteophyte formation, ligamental hypertrophy, and disc space reduction due to the pathological factors related to the instability, including weakness of paraspinal muscles, facetal telescoping, and “listhesis”^4^, ^9^. By contrast, extensive studies have indicated the importance of deep cervical flexors (DCFs), the longus capitis and colli, in support of the cervical lordosis and motion segments, and noted a close relationship between their impairments and neck pain^10^, ^11^, ^12^, ^13^, ^14^, ^15^. There exists an inferior ability to increase craniocervical flexion in individuals with neck pain, which results from the worse performance of DCFs^11^, ^13^, ^14^. Namely, the present data indicate that the changes in performance of craniocervical flexion test and clinical improvement may be because of changes in the DCFs^11^. This could be partly explained by the anatomical ground that the main action of DCFs is craniocervical flexion^15^. Thus, the craniocervical flexion test^11^ has been developed to assess the capacity of DCFs. Neck pain patients also performed less craniocervical flexion range of motion (ROM) to reach each pressure target of the craniocervical flexion test and this suggested that that the pressure increase in the muscle cuff under the cervical spine of neck pain patients was induced by a different movement strategy^11^, ^13^. Chronic neck pain has been demonstrated to be closely associated with CS^16^.

However, relevant research merely focused on neck pain and evaluated the ROM of craniocervical flexion using a digital camera and custom‐designed analytical software, which has limited accuracy when determining the real ROM. To our best knowledge, there are no prior studies that examine the role of atlanto‐occipital joint in the pathogenesis of CS. Whether the ROM of craniocervical flexion was associated with CS captured our interest, prompted both by the clinical observations that showed regular impairment of DCFs in patients with neck pain and by the functional anatomical research that confirmed their importance electromyographically in support of the cervical lordosis and motion segments. Currently, a need for flexion and even hyperflexion of the cervical segments has to increase, as many people use technological devices for recreation or/and work, especially smart phones for hours during the day. In our clinical observation, the craniocervical flexion angles vary within a relatively wide range from teenagers to the elderly. Therefore, it is of great necessity to clear the links between craniocervical flexion and CS.

The purpose of this study is as follows: (i) to measure the O‐C_1_ (atlanto‐occipital) angle from the lateral view and determine its interobserver and intraobserver reliability; (ii) to investigate the association between O‐C_1_ radiographic alignment in flexion and CS; and (iii) to discuss whether muscle dysfunction might play a role in preventing the normal functioning of the upper cervical spine. We hypothesized that stiff O‐C_1_, that is, flexion dysfunction of atlanto‐occipital joint, is closely associated with CS.

## Patients and Methods

### *Study Population*

CS patients were recruited from our hospital, and the age/gender/body mass index (BMI)‐matched healthy controls were selected from the subjects in health examinations at the same hospital between January 2015 and May 2019. According to the flexion function of the O‐C1 (FOC), the subjects were classified into low FOC and high FOC.

Inclusion criteria for the case group included: (i) the diagnosis of CS following the International Classification of Diseases 9th Revision (ICD‐9); (ii) receiving medical care at least three times during outpatient visits and/or one‐time hospitalization for principal/secondary diagnosis of CS; (iii) had completed data, including clinical evaluation and examination, that could be used for comparison; (iv) an observational study.

Exclusion criteria included: (i) those having congenital or acquired postural deformities such as kyphosis and scoliosis; (ii) spinal diseases such as tumors, fractures, and inflammation; (iii) headache resulting in daily activity limits; and (iv) history of any neck surgery.

Based on surgical treatments, CS patients were subdivided into non‐operation group and operation group. Operation group: patients with severe cervical spondylotic radiculopathy (CSR)/cervical spondylotic myelopathy (CSM), not improved by conservative treatment over a 12‐week observation; non‐operation group: the CS‐related symptoms can be improved by conservative treatment over a 12‐week observation. By contrast, subjects with no signs and symptoms of CS and no evidence of radiographic abnormalities were the control group. Every procedure performed in this study involving human subjects followed the ethical standards of the institutional review board of the hospital. All subjects were included after they provided informed written consent.

### *Surgical Technique*

All operations were performed by one surgical team using general anesthesia.

**Fig. 1:**
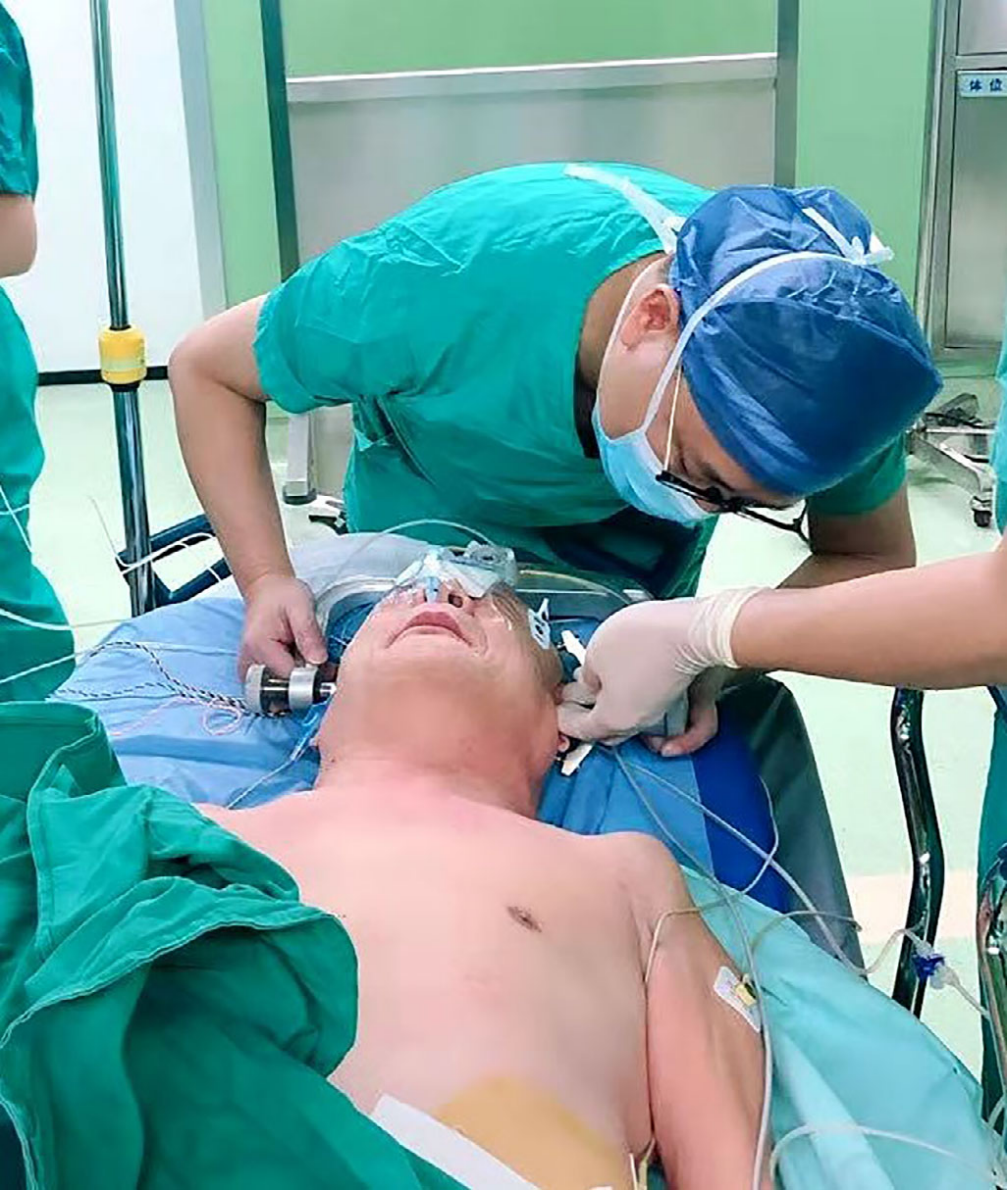
The Mayfield head frame was installed, and the cervical spine was fixed in flexion position.

**Fig. 2:**
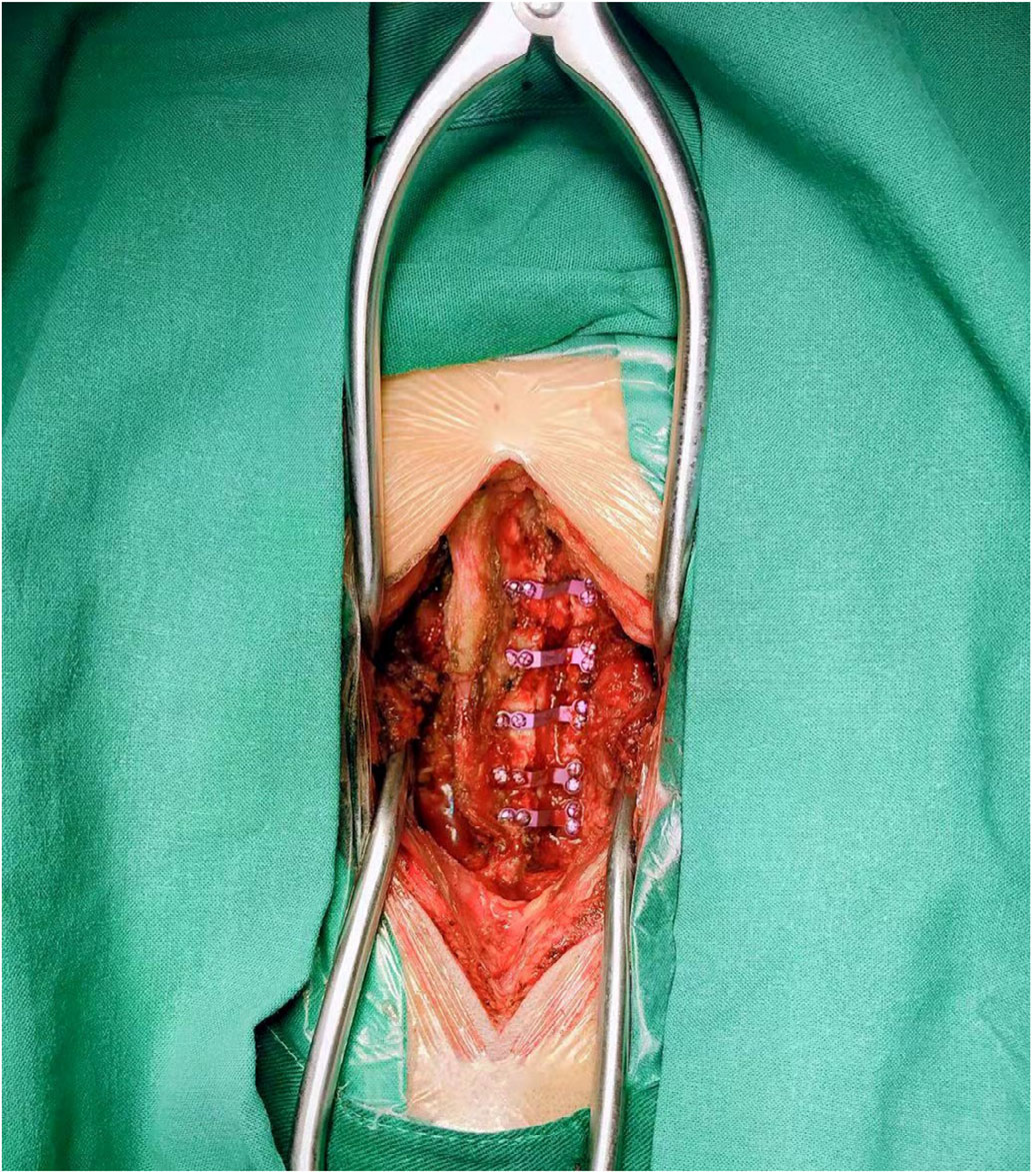
Supporting titanium plates (Centerpiece^TM^ Medtronic SofamorDanek, USA, Inc.) were inserted between the left lamina and the broken end of lateral mass.

The patient was placed in the prone position. The Mayfield head frame was installed (Fig. 1) and the cervical spine was fixed in flexion position. After the disinfection of the occipital and cervical region, the subcutaneous tissue from C_3_ to C_7_ was incised using the posterior middle approach, and the paravertebral muscles were carefully stripped to expose the lateral masses. The right lamina was used as the portal axis. The lateral cortex of the right lamina and the whole cortex of the left lamina were removed by bone biting forceps and ultrasonic osteotome. Lift lamina and spinous process to the right. We used 1 mm laminectomy forceps to trim marginal bone and remove parts of ligamentum flavum. Supporting titanium plates (Centerpiece^TM^ Medtronic SofamorDanek, USA, Inc.) were inserted between the left lamina and the broken end of lateral mass (Fig. 2). Each titanium plate was fixed by four screws. A drainage tube was placed.

Following the operation, the patients were arranged with X‐rays, computed tomography (CT), and/or magnetic resonance imaging (MRI) scan to monitor the decompression of the canal, alignment, and fusion. Besides, symptoms and physical examinations were used to assess the clinical outcomes.

### *Outcome Measures*

#### *Radiographic Evaluation*

##### The O‐C_1_ (Atlanto‐Occipital) Angle

**Fig. 3:**
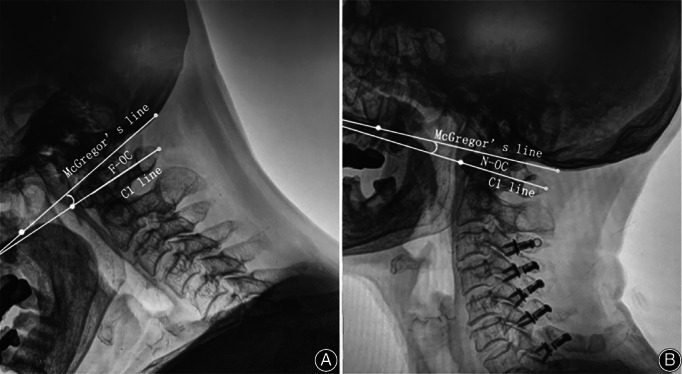
Representation of the radiographic measurement of O‐C_1_ angle in flexion position (A) and neutral (B) from the lateral view. The O‐C_1_ angle was measured between the McGregor's line and the line passing through the center of the C_1_ anterior arch and the center of the C_1_ posterior arch.

The O‐C_1_ (atlanto‐occipital) angle was measured on the lateral view (Fig. 3). This radiographic parameter was measured based on McGregor's line^7^ as the occipital baseline and the C_1_ line defined as the line between the centers of the anterior and posterior arch of the atlas. The O‐C_1_ angle was defined as the angle between McGregor's line and the C_1_ line. This angle reflected the ROM of O‐C_1_ joint.

##### F‐OC Angle

The O‐C_1_ angles were evaluated on images taken in flexion (F‐OC) (Fig. 3A). When taking a flexion view, the subject was required to the flexed cervical spine as much as possible. This angle indicates the flexion capacity of O‐C_1_ joint.

##### N‐OC Angle

The O‐C_1_ angles were evaluated on images taken in neutral positions (N‐OC) (Fig. 3B). The neutral position was defined as the subject looking forward and upright with the knee and hip extended. This angle indicates the extension capacity of O‐C_1_ joint.

##### FOC Angle

The flexion function of the O‐C_1_ (FOC) was defined as F‐OC minus N‐OC (Fig. 3). This angle indicates the maximum ROM of O‐C_1_ joint.

#### *Neck Disability Index*

Clinical outcome was evaluated by the Neck Disability Index (NDI) at first visit^16^. The NDI, a self‐reported questionnaire, quantifies neck‐related disability using a score from 0 (no disability) to 50 (maximum disability)^16^ and involves 10 daily activities, such as the ability to dress, lift heavy objects, read, work, and sleep. Higher scores represent a more severe disability.

### *Statistical Analysis*

The ordinal variables were described as proportions and quartiles. The chi‐square test for dichotomous variables was carried out to compare cases and controls. The Mann–Whitney, the Student's *t*‐test, or the Variance test was done for continuous variables. One‐way analysis of variance and a post‐hoc Tukey test was performed to compare FOC levels between the control and two case groups. To evaluate the reliability of techniques used to measure the O‐C_1_ angles, two authors assessed the intra‐ and interobserver variabilities. Each author measured a parameter twice, and then interobserver and intraobserver reliability was evaluated by calculating the intraclass correlation coefficient (ICC). The internal consistency of the measurements was characterized as excellent (ICC ≥ 0.9), good (0.7 ≤ ICC < 0.9), and acceptable (0.6 ≤ ICC < 0.7)^17^. The Pearson correlation coefficient was calculated to find a correlation between FOC and NDI. Receiver operator curve (ROC) analysis by calculating the area under the curve was performed to estimate the cut‐off values to determine FOC's predictive role in CS. The risk analysis model by logistic regression was performed to estimate the odds ratios (*OR*s) and the associated 95% confidence interval (*CI*) to identify factors independently associated with CS. Multivariate logistic models were performed using stepwise elimination of variables of interest from univariate analysis. The statistical significance and power analysis were set at *P* value ≤0.05 and 0.8. All analyses were performed using SPSS version 20.0 (SPSS; Chicago, IL, USA).

## Results

### *Subject Characteristics*

A total of 464 subjects was included in the study with the median follow‐up time of 56.6 months (25–121 months). The operation group and non‐operation group had 45 and 187 patients, respectively, while 232 subjects were included in the control group. The characteristics of the subjects are listed in Table 1.

**TABLE 1:** Subject characteristics

Groups	Male (*n*, %)	Age (years, Mean ± SD)	BMI (kg/m^2^, Mean ± SD)	NDI	Type of CS (cases [%])		Medical history of CS (years, Mean ± SD)
					CSR	CSM	
Operation group (*n* = 45)	26 (57.8%)	55.8 ± 11.2	22.5 ± 4.2	12.2 ± 4.5	34 (75.6%)	11 (24.4%)	10.5 ± 9.5
Non‐operation group (*n* = 187)	116 (62.0%)	41.6 ± 13.8	23.2 ± 3.8	6.2 ± 2.1	175 (93.6%)	12 (6.4%)	6.8 ± 11.2
Control group (*n* = 232)	140 (60.0%)	23.5 ± 5.5	23.5 ± 4.5	3.2 ± 1.2	—	—	—
*P* value	0.8558	<0.001	0.3249	<0.001	<0.001	<0.001	<0.001

BMI, Body Mass Index; CSM, Cervical Spondylotic Myelopathy; CSR, Cervical Spondylotic Radiculopathy; NDI, Neck Disability Index.

There were no significant differences in gender (male: 57.8% *vs* 62.0% *vs* 60.0%, *P* = 0.8558) and BMI between groups (22.5 ± 4.2 *vs* 23.2 ± 3.8 *vs* 23.5 ± 4.5 kg/m^2^, *P* = 0.3249). However, the case groups, especially the operation group, tended to be older (55.8 ± 11.2 *vs* 41.6 ± 13.8 *vs* 23.5 ± 5.5 years, *P* < 0.001) and have a higher NDI score (12.2 ± 4.5 *vs* 6.2 ± 2.1 *vs* 3.2 ± 1.2, *P* < 0.001). In the operation case group, there was more patients with CSM (24.4% *vs* 6.4%, *P* < 0.05) and longer medical history of CS (10.5 ± 9.5 *vs* 6.8 ± 11.2 years, *P* < 0.001) (Table 1).

### *Difference of Flexion Function of O‐C1 (FOC) Among Groups*

One‐way analysis of variance showed statistically significant differences in FOC between the control and case groups (1.4° ± 1.2° *vs* 3.6° ± 1.9° *vs* 7.2° ± 2.0°, *P* < 0.001) (Table 2). Besides, a post‐hoc Tukey test showed a lower FOC in the operation group compared with that in the non‐operation group (1.4° ± 1.2° *vs* 3.6° ± 1.9°, *P* < 0.001). N‐OC and F‐OC were not statistically significantly different among the three groups (11.0° ± 4.2° *vs* 10.0° ± 4.0° *vs* 10.2° ± 4.6°, *P* = 0.1373; 12.4° ± 4.0° *vs* 13.6° ± 4.2° *vs* 17.4° ± 4.5°, *P* = 0.0839, respectively).

**TABLE 2:** Comparison of Flexion Function of O‐C1 (FOC) among groups (Mean ± SD)

Groups	FOC	N‐OC	F‐OC
Operation group (*n* = 45)	1.4 ± 1.2	3.6+1.9	7.2 ± 2.0
Non‐operation group (*n* = 187)	11.0 ± 4.2	10.0 ± 4.0	10.2 ± 4.6
Control group (*n* = 232)	12.4 ± 4.0	13.6 ± 4.2	17.4 ± 4.5
*P* value	<0.001*	0.1373	0.0839

FOC, Flexion Function of O‐C_1_; F‐OC, O‐C_1_ angles in flexion; N‐OC, neutral positions.

### *A Predictive Model of FOC as a Radiological Marker for CS*

**Fig. 4:**
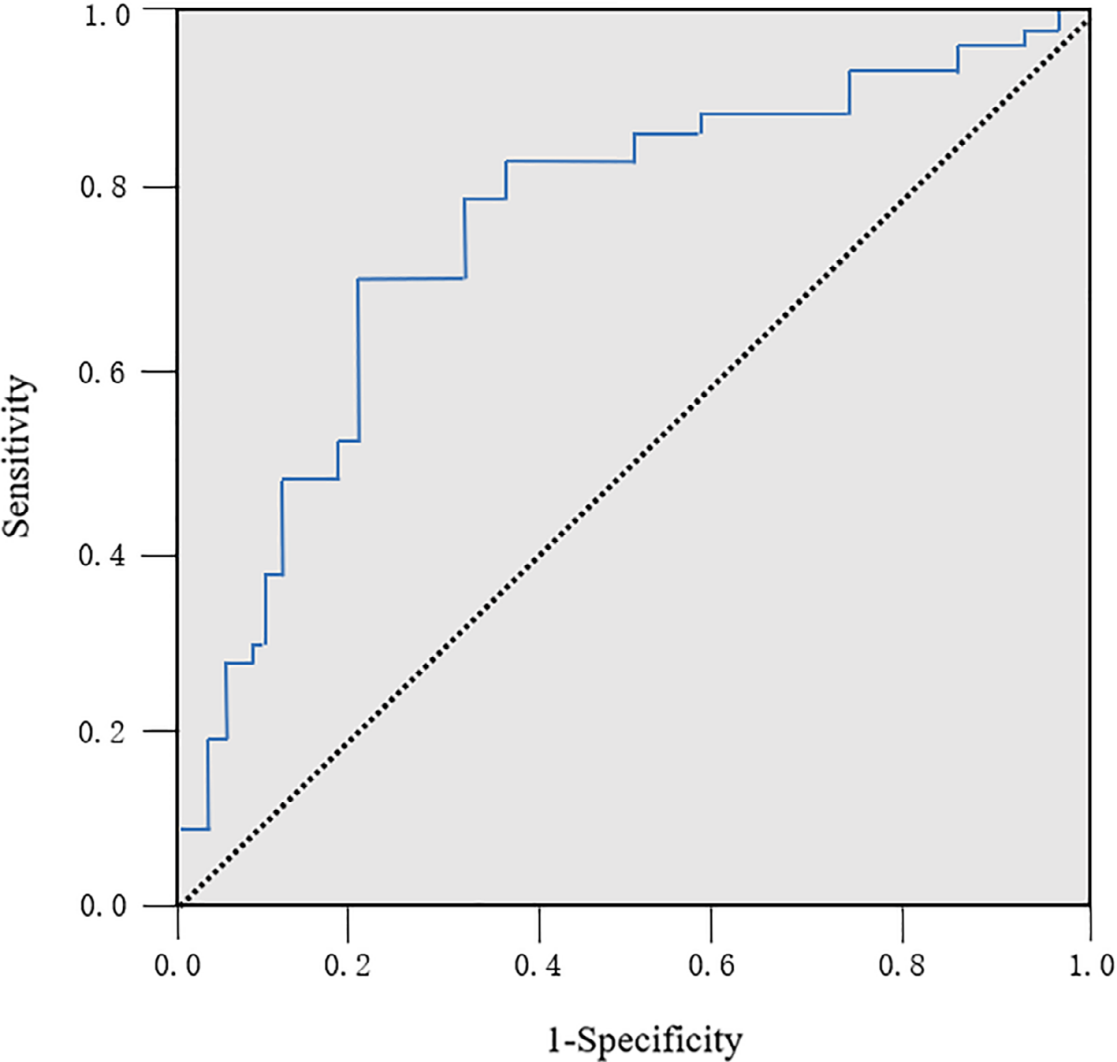
Receiver operating curve (ROC) of FOC. The area under the curve (AUC) was 0.86. FOC had a sensitivity, specificity, Youden index, and cut‐off value of 87%, 90%, 0.77, and 4.2°, respectively.

Using FOC as a radiological predictive model to predict CS, the area under the curve (AUC) was 0.86 (95% CI: 0.78–0.92, *P* < 0.001) (Fig. 4). FOC had a sensitivity, specificity, Youden index, and cut‐off value of 87%, 90%, 0.77, and 4.2°, respectively.

### *Subgroup Analysis for Low FOC and High FOC*

According to the cut‐off value of 4.2°, 152 subjects (32.8%) had low FOC and 312 (67.2%) had high FOC.

There were no significant differences in age (49.9 ± 12.2 *vs* 53.2 ± 13.0, *P* = 0.009), gender (male: 53.5% *vs* 56.8%, *P* = 0.548), and BMI (22.8 ± 4.0*vs* 23.4 ± 3.9, *P* = 0.1237) between low FOC group and high FOC group. However, the low FOC group had a higher NDI score in comparison with high FOC group (8.3 ± 3.0 *vs* 5.1 ± 2.7, *P* < 0.001) and tended to experience more risks for CS (63.1% *vs* 43.6%, *P* < 0.001) and operation (14.5% *vs* 7.4%, *P* = 0.024).

### *Risk Analysis Model for FOC in CS*

Risk analysis was based on the cut‐off value calculated by ROC, with those higher than 4.2° classified as having a better FOC. In the univariable risk analysis model, conditional logistic regression showed that the FOC level was an independent factor with an important role in the risk of CS. The odds rose 8.2 times (*OR* = 8.2; 95% *CI*: 6.4–10.0; *P* < 0.001) when FOC reached the level under the 4.2°. After adjusted for gender, age, and BMI, the multivariate risk analysis model revealed that only FOC level (*OR* = 6.5; 95% *CI*: 5.2–7.8; *P* < 0.001) remained a risk factor for the CS.

### *Distribution of Low‐Level FOC in Different Age Groups*

**Fig. 5:**
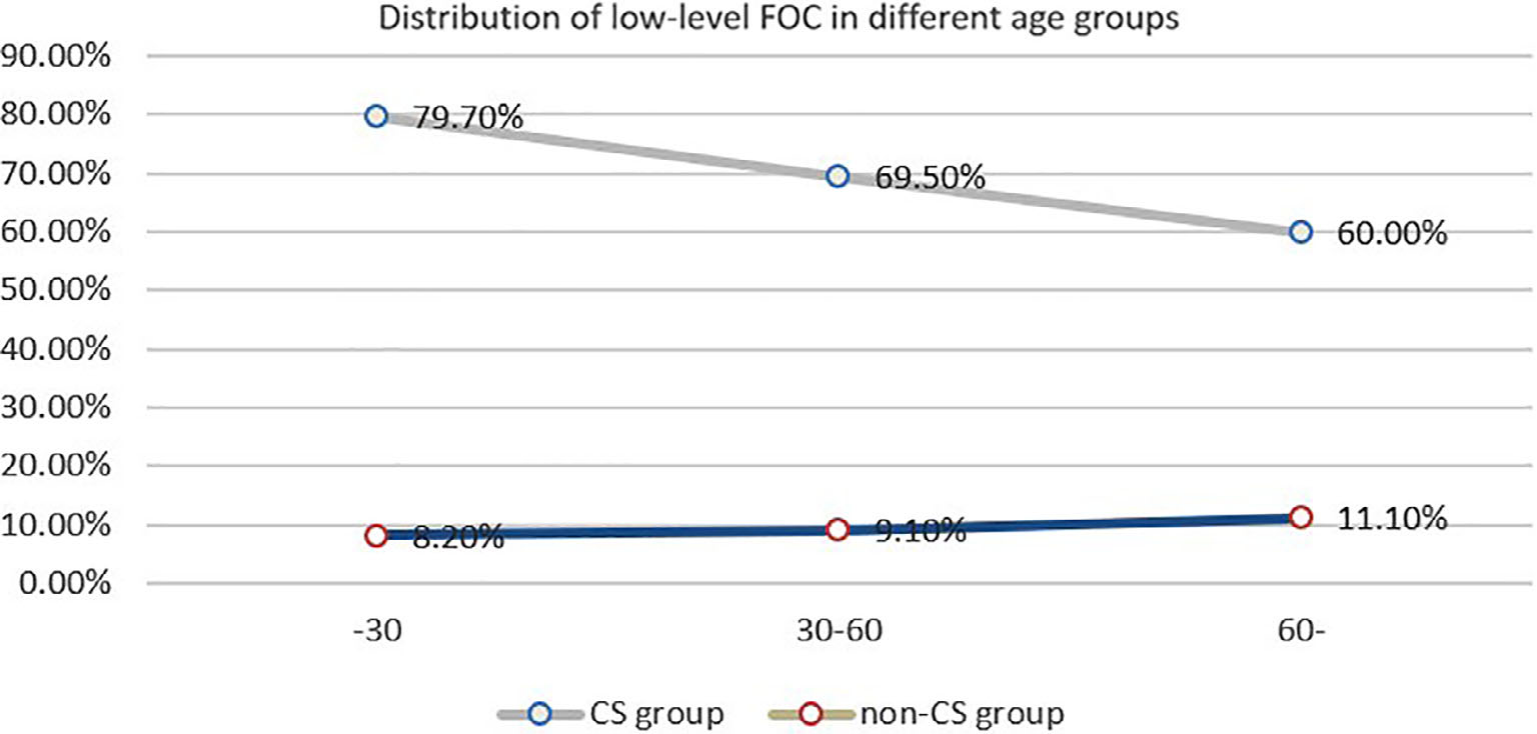
Distribution of low FOC in different age groups. The percentage of individuals with a FOC level of less than 4.2° slightly increased with the increasing age in the cohort of the non‐CS population. However, the percentage of their counterparts in the cohort of the CS population experienced an obvious decrease with increasing age from 79.7% to 60.0%.

The percentage of individuals with a FOC level of less than 4.2° slightly increased with the increasing age in the cohort of the non‐CS population. However, the percentage of their counterparts in the cohort of the CS population experienced an obvious decrease with the increasing age from 79.7% to 60.0% (Fig. 5).

### *Correlation Between FOC Levels and NDI*

**Fig. 6:**
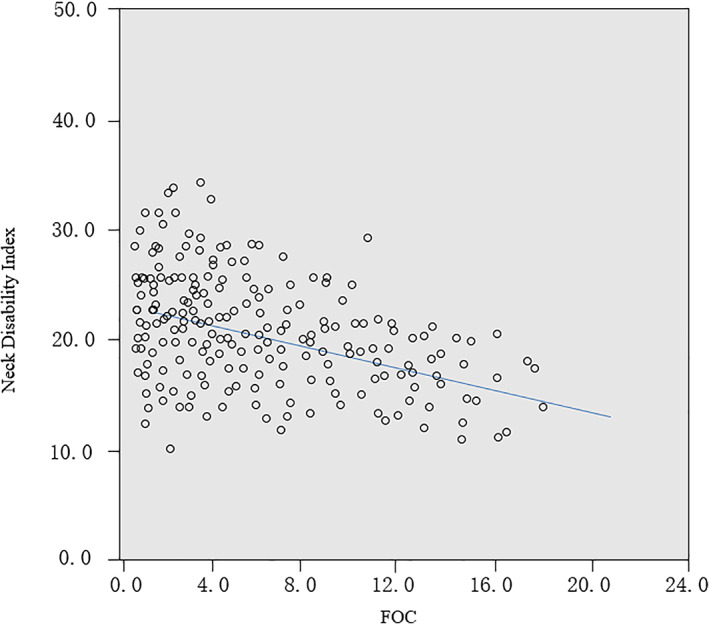
A significant negative correlation between the FOC and NDI for CS patients by controlling the variables of age, gender, BMI (*Y* = 22.343 − 0.451*X*, *R* ^2^ = 0.203, *P* = 0.016).

A correlation test was performed using a partial correlation test only for CS patients by controlling the variables of age, gender, and BMI to determine the correlation between FOC levels and NDI. There existed a significant negative correlation between FOC levels and NDI (*r* = −0.451; *P* = 0.016) (Fig. 6).

### *Reliability Analysis of ROC Measurements*

The results for FOC showed good interobserver reliability (ICC = 0.865) and excellent intra observer reliability (ICC = 0.911).

### *Case Presentation*

**Fig. 7:**
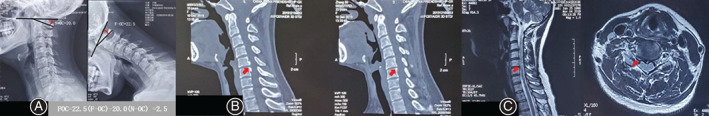
A 29‐year‐old female with a stiff atlanto‐occipital joint. (A) Straightening of the physiologic lordosis and degeneration of cervical segments, and decreased FOC in the atlanto‐occipital joint. (B) CT reveals sclerosis of the vertebral body edge and facet joint (red arrow). (C) Straightening of the physiologic lordosis and degeneration of cervical segments, MRI T2‐weighted axial imaging at the level of C_5‐6_ showing right side C_6_ nerve root compression (nerve root sleeve disappearance, red arrow) in addition to the spinal cord compression.

A 33‐year‐old female teacher had had intermittent pain in her neck and shoulder and numbness in the medial side of her right upper arm for one year. On admission, she had experienced aggravated pain for 2 months in addition to the paralysis of her right upper extremity. The X‐ray showed a stiff atlanto‐occipital joint (FOC = 2.5°) (Fig. 7A) and CT examination showed sclerosis of the vertebral body edge and facet joint (Fig. 7B). The MRI T2‐weighted sagittal imaging revealed dural sac compression from C_3_ to C_7_ with a C_5‐6_ herniated disc and spinal cord anterior indentation at the level of C_5‐6_ with a high signal change. MRI T2‐weighted axial imaging at the level of C_5‐6_ showed right side C_6_ nerve root compression in addition to the spinal cord compression (Fig. 7C). She accepted the plate and screwed internal fixation with an anterior discectomy.

## Discussion

Previous studies indicated that pathophysiological changes, such as disc degeneration and disc protrusion, were surprisingly common findings in young adults with neck pain^18^, ^19^. The course and outcome of CS are highly varied, especially in young patients^18^. A minority of them have to accept surgical treatments due to severe pain and neurologic symptoms, while others do not. Several studies investigated anatomical variables of the cervical vertebrae as risk factors, such as anteroposterior vertebral body diameter, mid‐sagittal vertebral canal diameter, and the canal–body ratio^19^, ^20^. However, these anatomical variations just statically reveal their effects.

CS gradually occurs over time, accompanied by dynamic activities that are complex anatomically and biomechanically. Cheng Huang reported that in a population of 10,930 dentists and 73,718 controls, younger dentists had a higher risk of developing cervical herniated intervertebral disc^19^. Sustained contraction of the cervical muscles to keep forward‐head postures, which involve holding the neck and head in an unbalanced and unnatural forward position to gain better visibility, is universally unavoidable in daily dental operations^21^. The painless, insidious nature of repetitive minor trauma caused by such a prolonged static posture (PSP) may lead to the degeneration of spinal discs^19^, ^21^, ^22^. These suggested that there existed a significant role of the head's movement in a pathophysiologic change of the cervical spine. This effect is produced through the craniovertebral junction (CVJ), including the atlanto‐occipital joint and its surrounding ligaments and muscles. DNFs are the only cervical muscles that lie close in front of the cervical spine and have attachments confined to the vertebrae^22^. DNFs, which function as cervical segmental flexors that provide physical support to the cervical column and dynamically stabilize the neck, are considered necessary for the cervical spine^11^, ^12^, ^13^, ^22^. A compromised function is a feature of neck pain disorders, including whiplash‐induced, idiopathic, work‐related neck pain^12^, ^13^, ^14^, ^15^. Retraining DCFs has been shown to decrease neck pain and improve the ability to maintain an upright posture of the cervical spine^14^, ^15^, ^16^.

The force initiated from the head is transferred to the lower segments by the atlanto‐occipital joint. Therefore, stiff atlanto‐occipital joints, represented by low FOC, increase mechanical stress downwards. This may aggravate degeneration or herniation of the spinal discs^19^, ^23^. The presented case (Fig. 5) with a limited FOC presented with obvious degenerative changes in cervical segments and this further supported our present assumption. However, our results seemingly contradict previous similar studies. For example, Hayashi *et al*.^24^ found that decreased subaxial cervical spinal motion is associated with intervertebral disc degeneration in the asymptomatic population. This decrease in mobility at the subaxial cervical spine is compensated for by an increase in angular mobility of the upper cervical spine at the occipital‐atlantoaxial complex, especially at O‐C_1_. Our results showed that despite degeneration in the subaxial cervical spine for younger patients (Fig. 5), they tend to have a stiffness O‐C_1_ instead of compensatory increased O‐C_1_. As for such a contradiction, we deemed that Hayashi *et al*. included symptomatic patients with an average age of 50.2 years in a rather wide range (range, 19–79 years), while in our study, we classified CS according to different severities and ages with healthy controls in order to obtain more information. This method allowed us to understand that the etiology of CS is various and complicated. Our results revealed that the low FOC group had a higher NDI score in comparison with high FOC group and tended to experience more risks for CS and operation. Therefore, we believe that the middle‐lower cervical segments and the upper one must interact with each other in a natural and intricate biomechanical balance. A biomechanical study about their relationship is required.

Our results showed that the gap for the percentage of low FOC in three different age groups between cases and controls tended to be narrowed as the age increased (Fig. 3). Biological elements such as degenerated discs and ligaments gradually appear, reveal themselves, and ultimately develop into CS^4^. However, it is unusual to see these in young people. Hence, the larger gap in the 30‐year‐old group may help indicate the negative role of low FOC in the development of CS.

There existed a significant negative correlation between FOC and NDI (*r* = −0.451, *P* = 0.016) (Fig. 4). Mousavi‐Khatir *et al*. reported that a 10‐min static flexion could lead to changes in the neck proprioception and feed‐forward control on account of mechanical and neuromuscular changes in the viscoelastic cervical spine structures, and these changes in sensory‐motor control could be a risk factor for neck pain and injury^25^. Besides, PSPs are much more taxing than moving forces^26^. Suffering from PSPs, the subsequent consequences of neck muscular tissues include muscle fatigue, protective muscle contraction, muscle imbalance, and, finally, spinal disc degeneration or herniation^26^. In light of the transferring role of CVJ, the stiff atlanto‐occipital joint could make the middle‐lower cervical segments experience more PSPs. In this way, FOC levels were negatively correlated with NDI.

This is the first study to demonstrate that the lower flexion function of the atlanto‐occipital joint could be a risk for accelerated degeneration of CS. The implication of this study lies in the understanding that individuals with low FOC can be screened by a cheap, easily accessible X‐ray. These people should receive more education and care about musculoskeletal health related to the prevention of CS as early as possible. Currently, a need for flexion and even hyperflexion of the cervical segments has to increase, as many people use technological devices for recreation or/and work, especially smart phones for hours during the day. In 2014, the proportion of adolescents who used the smart phone was more than 60% in Shanghai, China, and this figure still keeps rising^27^. Therefore, considering the adverse health effects of the increasing prevalence of smart phones on young adults, the present risk analysis of FOC due to such exposure could be a promising new parameter in predicting CS, which is of great significance to spine surgeons.

## Limitation

The present study had a few limitations. First, the primary limitation is its retrospective case‐control design, which meant it was difficult to deduce causality from the results. A prospective study and biomechanical experiments are needed to support our findings. Second, this is a retrospective study based on X‐ray instead of CT, which reflects the real FOC in a more accurate way. Although X‐ray is not the best choice to depict FOC, we identified the bone cortex as best as we could, and, meanwhile, our results revealed that X‐ray was also practical to detect individuals with low FOC. Last, there was inconsistency regarding the atlanto‐occipital angles in flexion and neutral positions between our results and those of Dohzono^23^. Dohzono reported that the atlanto‐occipital angle decreased when the neck flexed from the mean of 12.4° in the neutral position to 8.6° in flexion. However, our result showed the opposite situation where the angle increased (Table 2). These limitations open the door for future studies.

## Conclusion

Patients with CS tend to have stiff O‐C_1_. Muscle dysfunction might play a role in preventing the normal functioning of the upper cervical spine. This study showed that FOC less than 4.2° could experience an 8.2‐fold increase in the odds of CS. This finding could help spine surgeons to understand the pathological process of CS and implement appropriate management.
